# Decoding Kidney Pathophysiology: Omics-Driven Approaches in Precision Medicine

**DOI:** 10.3390/jpm14121157

**Published:** 2024-12-19

**Authors:** Charlotte Delrue, Marijn M. Speeckaert

**Affiliations:** 1 Department of Nephrology, Ghent University Hospital, 9000 Ghent, Belgium; charlotte.delrue@ugent.be; 2 Research Foundation-Flanders (FWO), 1000 Brussels, Belgium

**Keywords:** multi-omics, precision medicine, kidney disease biomarkers

## Abstract

Chronic kidney disease (CKD) is a major worldwide health concern because of its progressive nature and complex biology. Traditional diagnostic and therapeutic approaches usually fail to account for disease heterogeneity, resulting in low efficacy. Precision medicine offers a novel approach to studying kidney disease by combining omics technologies such as genomics, transcriptomics, proteomics, metabolomics, and epigenomics. By identifying discrete disease subtypes, molecular biomarkers, and therapeutic targets, these technologies pave the way for personalized treatment approaches. Multi-omics integration has enhanced our understanding of CKD by revealing intricate molecular linkages and pathways that contribute to treatment resistance and disease progression. While pharmacogenomics offers insights into expected responses to personalized treatments, single-cell and spatial transcriptomics can be utilized to investigate biological heterogeneity. Despite significant development, challenges persist, including data integration concerns, high costs, and ethical quandaries. Standardized data protocols, collaborative data-sharing frameworks, and advanced computational tools such as machine learning and causal inference models are required to address these challenges. With the advancement of omics technology, nephrology may benefit from improved diagnostic accuracy, risk assessment, and personalized care. By overcoming these barriers, precision medicine has the potential to develop novel techniques for improving patient outcomes in kidney disease treatment.

## 1. Introduction

Chronic kidney disease (CKD) is a major global health problem that raises the risk of morbidity and mortality. CKD is characterized by a slow decrease in kidney function over time and often leads to end-stage kidney disease (ESKD). The underlying causes of these diseases are complex and involve different cellular and molecular processes, such as inflammation, oxidative stress, and scarring [1]. Advances in omics technologies, such as genomics, transcriptomics, proteomics, and metabolomics, have increased our understanding of the mechanisms behind kidney diseases. The integration of multi-omics data makes it easier to discover new biomarkers and therapeutic targets, potentially leading to more tailored and successful treatments [2]. Despite these advances, applying precision medicine in nephrology still presents hurdles, such as data integration issues, ethical constraints, and the requirement for large-scale, high-quality datasets. Addressing these problems is critical for transferring omics-driven insights into clinical practice and thereby improving patient outcomes in kidney disease care. The purpose of this review is to provide an in-depth analysis of the use of omics technologies in nephrology, highlighting their potential for advancing precision medicine in the diagnosis, treatment, and management of CKD as well as to discuss the challenges and future directions of incorporating multi-omics data into clinical practice.

## 2. Omics Technologies in Kidney Research

### 2.1. Genomics

Genomic studies have greatly improved our understanding of kidney disease by finding genetic variants that contribute to its development (Table 1). High-throughput sequencing technology has identified many variants linked to various kidney diseases. Single gene mutations can result in monogenic kidney diseases. For example, mutations in the *PKD1* and *PKD2* genes are the main cause of autosomal dominant polycystic kidney disease (ADPKD), which leads to cyst formation and gradual kidney failure. About 85% of ADPKD cases are caused by *PKD1* mutations, whereas 15% are caused by *PKD2* mutations. Recent findings of new mutations in these genes have improved diagnosis precision [3,4]. Recent research has extended the mutation range of these genes, increasing diagnostic precision. The *COL4A3*, *COL4A4*, and *COL4A5* genes are mutated in Alport syndrome. The symptoms of this disease include glomerulonephritis, hearing loss, and vision issues. Mutations affecting the production and assembly of type IV collagen α3α4α5 heterotrimers can compromise the structural integrity of the basement membranes in the kidneys, cochlea, and eyes. Recent expansions in the number of known pathogenic variants in these genes have improved our knowledge of the connections between genotype and phenotype as well as the accuracy of diagnostics [5,6]. Advances in genome sequencing have made it easier to discover these mutations, allowing for early diagnosis and therapy [7]. In addition to monogenic disorders, complex kidney diseases are caused by interactions between multiple genetic variants and environmental factors. Genome-wide association studies (GWAS) have identified a large number of loci associated with CKD susceptibility. Notably, mutations around the uromodulin-encoding *UMOD* gene have been linked to a higher risk of CKD. Additionally, rare *UMOD* mutations can cause autosomal dominant tubulointerstitial kidney disease, further implicating uromodulin in renal pathology [8,9]. These mutations influence kidney function and disease progression because they are associated with increased uromodulin expression [7]. In some communities, some genetic variants increase the risk of kidney disease. Variants in the *APOL1* gene, for example, are common in African populations and have been associated with an increased risk of hypertension-related nephropathy and focal segmental glomerulosclerosis (FSGS). These risk variations, known as G1 and G2, have been found to be significant contributors to the renal disease burden in these populations. The presence of two *APOL1* risk alleles greatly increases the risk of various renal disorders [10,11]. Recent studies [12,13,14] have shown that around one-third of people in West African nations contain *APOL1* risk alleles, underscoring the need for genetic studies in this particular group [15]. Thanks to functional genomics, we now have a better understanding of the pathways affected by these genetic variants. To investigate the molecular processes impacted by mutations, including apicobasal polarity and cell–cell junctions, researchers employed kidney organoids produced from patient-induced pluripotent stem cells (iPSCs). These findings establish a solid framework for understanding the impact of genetic variants outside the core sequence [16]. Furthermore, many of the genes discovered in CKD GWAS, including *Dab2*, are required for tubular function and CKD progression, making them prospective therapeutic targets [17].

Genetic testing for harmful mutations improves prognosis and treatment regimens. For example, the discovery of *APOL1* risk polymorphisms may have an impact on kidney transplantation and hypertensive nephropathy therapy. In kidney transplantation, the *APOL1* genotypes of both the donor and the recipient can influence allograft results. Regardless of the donor’s *APOL1* status, recipients with high-risk *APOL1* genotypes may experience higher rates of T cell-mediated rejection and shorter allograft survival times. Additionally, kidneys from donors with two *APOL1* risk alleles have been associated with a worse transplant survival rate. These data indicate that *APOL1* genotyping may be useful in donor selection and post-transplant care. *APOL1* risk polymorphisms are associated with an increased vulnerability to hypertension-related CKD. Understanding a patient’s *APOL1* status may help to develop individualized therapy methods, perhaps leading to the better management of hypertensive nephropathy [28,29]. Furthermore, understanding the genetic basis of kidney diseases enables the development of customized medicines, such as the study of inaxaplin (VX-407) for treating ADPKD in persons carrying specific *PKD1* mutations [30,31,32,33]. Furthermore, polygenic risk scores (PRS) have emerged as a promising method for combining the impacts of several genetic variants, improving risk assessment, and encouraging early interventions in genetically predisposed populations [18].

### 2.2. Transcriptomics

Transcriptomics, or the comprehensive study of RNA transcripts within cells or tissues, is an effective tool for understanding the molecular origins of renal problems. In this field of study, researchers may analyze the expression of thousands of genes at the same time, providing insight into cellular activity and relationships in both healthy and diseased states. By examining the transcriptome, or the complete set of RNA molecules in a single cell or tissue, researchers can discover genes that are differentially expressed, discover novel biomarkers associated with kidney disease, and obtain a better understanding of complex biochemical processes. For example, transcriptome analysis has identified new molecular biomarkers involved in the inflammatory processes of kidney disorders such as IgA nephropathy and lupus nephritis. These findings increase our understanding of the mechanisms behind kidney disease and pave the way for new diagnostic and treatment approaches [34,35].

Single-cell RNA sequencing (scRNA-seq), a revolutionary transcriptomics approach, measures gene expression at the individual cell level to learn about the cellular diversity of kidney tissue. In contrast to conventional bulk RNA sequencing, scRNA-seq can identify new cell populations implicated in kidney pathology, development, and function in addition to subtle variations among cell types. ScRNA-seq, for example, has been used to classify cells in the adult mouse kidney into 16 clusters, which include 13 known cell types and three unknown populations. Furthermore, the scRNA-seq of human kidneys revealed 13 clusters of normal renal cells, resulting in a complete cellular atlas [36]. Furthermore, scRNA-seq has identified unique immune cell subsets involved in inflammation and autoimmunity in disorders such as lupus nephritis, offering insights into the disease’s underlying mechanisms. In kidneys affected by lupus nephritis, there is an increase in CD163^+^ dendritic cells, which are pro-inflammatory and interact with CD4^+^ T cells, resulting in local immunological activation [37]. ScRNA-seq has revealed the presence of atypical B cells and antibody-secreting cells within the renal tissue of patients with LN, indicating an active extrafollicular B cell response that may drive disease progression [38]. By highlighting cell-specific transcriptional changes, single-cell transcriptomics has become a crucial tool for mapping cellular interactions and pinpointing cell types that drive renal disease progression [20]. Non-coding RNAs, such as long non-coding RNAs (lncRNAs) and microRNAs (miRNAs), are increasingly understood to have regulatory functions in kidney disease. LncRNAs can affect renal fibrosis by interacting with the TGF-β signaling pathway, a key fibrosis regulator. Additionally, various miRNAs, including miR-21-5p and miR-29a/b/c, have been linked to the development and progression of renal fibrosis. These non-coding elements affect the expression of genes necessary for kidney health that are engaged in fibrotic, inflammatory, and metabolic processes [39,40,41]. For instance, miRNAs are interesting therapeutic targets since they have been linked to pathological processes such as fibrosis in diabetic kidney disease (DKD). MiR-21 is highly elevated in DKD and has a role in renal fibrosis by enhancing extracellular matrix buildup and epithelial-mesenchymal transition. Antisense oligonucleotides that target miR-21 have been shown to improve renal function and lessen fibrosis in DKD experimental animals. The reduced expression of the miR-29 family has been found to increase collagen synthesis in DKD, making it a negative regulator of fibrosis. One potential treatment approach for lowering renal fibrosis is to restore miR-29 levels [42,43]. Previously unannotated miRNAs have been identified in kidney tissues, revealing their potential diagnostic and prognostic value, especially in cancers like clear cell renal cell carcinoma (ccRCC). A study discovered nine dysregulated miRNAs in ccRCC tissues compared to normal renal parenchyma, indicating their potential as disease indicators. Several miRNAs, most notably Knm22_2209 and Knm6_2419, have been linked to patient survival, suggesting their potential as disease markers [44]. Target prediction studies have shown that these miRNAs are also engaged in important pathways such as axon guidance, mitogen-activated protein kinase (MAPK) signaling, and vascular endothelial growth factor (VEGF) signaling. MiR-132 promotes axon outgrowth by reducing Rasa1, an inhibitor of the MAPK/ERK and PI3K signaling pathways [45]. Understanding these pathways is critical for understanding renal pathology and evaluating treatment choices [46].

Spatial transcriptomics (ST) combines gene expression profiling with spatial context to preserve the architecture of kidney tissue during analysis. This approach has changed our understanding of how cells interact in their natural settings by directly mapping gene expression patterns onto tissue slices. ST has been used in kidney studies to analyze cellular organization within nephron segments as well as to detect localized differences in gene expression associated with diseases. For example, ST enables researchers to investigate how cells in the tubule microenvironment communicate in response to genetic changes or environmental stresses. ST enables us to visualize the spatial distribution of cellular responses, which aids our understanding of how tissue shape influences kidney function and disease. ST has permitted the construction of spatially anchored human kidney atlases in both healthy and sick tissues, contributing to our understanding of the cellular and molecular pathways behind kidney disorders [47,48]. In addition, population-specific transcriptome diversity is important in understanding kidney disease risk. Transcriptomic investigations across ethnicities, for example, have revealed diversity in gene expression related with disease vulnerability, such as *APOL1*-associated kidney disease in African populations [49]. These findings emphasize the necessity for population-specific research to discover risk factors that may be unique to certain genetic origins [50].

### 2.3. Proteomics

Proteome analysis examines dynamic changes in response to disease to identify molecular cues that genomes and transcriptomics may miss. More specifically, protein expression levels, protein–protein interactions, post-translational modifications (PTMs), and protein localization have been investigated. PTMs such as phosphorylation and glycosylation can significantly alter protein activity, localization, and interactions, thereby influencing disease progression. Recent research has highlighted the role of PTMs in altering protein–protein interactions and cellular signaling cascades, emphasizing their significance in both health and illness [51]. Quantitative proteomics, which includes techniques like mass spectrometry and antibody-based assays, has made it easier to discover proteins that are differentially expressed in diverse kidney disorders. CKD has been associated with protein expression patterns related to inflammation, fibrosis, and oxidative stress. These profiles provide insight into the underlying causes of CKD, in addition to potential treatment targets for slowing or even reversing the disease [22,52]. Certain proteome patterns have been found to be important in the genesis of CKD, especially those related to the ubiquitin–proteasome pathways and the complement cascade. Reduced proteasome activity and complement system overactivation have been associated with chronic inflammation and fibrosis, both of which are significant hallmarks of CKD progression. These processes are emphasized by biomarkers such as biglycan, transthyretin, and complement component C6, which also represent potential treatment targets for increasing proteasome activity or changing complement activation, providing promising approaches to slow disease progression [22]. Analyses of temporal and spatial proteomics, like transcriptomics, have been crucial in monitoring the course of disease over time and between kidney compartments. By mapping protein distributions within kidney tissue slices, for instance, spatially resolved proteomics has allowed researchers to gain insight into localized pathophysiological alterations in response to damage [53] and to identify the crucial time for intervention [54]. Understanding protein–protein interactions and functional networks is critical in kidney diseases, regardless of individual protein expression levels. Network-based analysis has revealed key proteins, such as connective tissue growth factor (CTGF), that regulate fibrotic processes in CKD. CTGF interacts with signaling molecules like TGF-β to cause extracellular matrix formation and tissue scarring. Network-based studies aid in identifying hubs and modules associated with inflammation and fibrosis, two processes crucial to the course of CKD [55]. Key proteins and processes that contribute to the molecular framework of DKD, such as inflammatory cascades, have been discovered by such interaction studies [56]. Autophagy and the ubiquitin–proteasome system are two protein degradation mechanisms that are essential for maintaining cellular homeostasis in kidney disorders. The ubiquitin–proteasome system preferentially destroys damaged or misfolded proteins, preventing buildup and cytotoxicity. Autophagy, on the other hand, helps to degrade bigger cellular components, such as organelles, via lysosomal pathways. A failure in either of these systems might result in the accumulation of damaged proteins and organelles, exacerbating kidney disease. For example, decreased ubiquitin–proteasome system activity has been associated with renal fibrosis and other kidney disorders. Similarly, faulty autophagy has been linked to a variety of kidney illnesses, emphasizing its importance in renal health. The interaction between autophagy and the ubiquitin–proteasome system is critical for cellular quality control, and their failure can accelerate kidney disease progression [57,58,59]. In diseases like DKD and CKD, disruptions in these pathways have been linked to cellular stress and protein buildup in glomerular and tubular cells [23]. PTMs like phosphorylation, glycosylation, acetylation, and ubiquitination can affect a protein’s activity, stability, location, and interactions with other molecules, thereby influencing biological processes and reactions. Proteomic studies in kidney disease research have found alterations associated with disease states by characterizing PTMs in kidney tissues and fluids. These PTM alterations may play an important role in the pathophysiology of the disease, as evidenced by the discovery of aberrant phosphorylation patterns in signaling proteins implicated in kidney fibrosis and the association of CKD with the aberrant glycosylation of uromodulin. Abnormal phosphorylation patterns in signaling proteins, notably those implicated in the epidermal growth factor receptor (EGFR) pathway, are linked to renal fibrosis, which marks the course of CKD. The overactivation of EGFR signaling promotes fibrogenesis, implying that dysregulated phosphorylation events play an important role in the process. Furthermore, CKD has been linked to changes in glycosylation, specifically of uromodulin. The most prevalent protein in normal urine, uromodulin, passes through particular glycosylation patterns that are necessary for it to function. Alterations in these glycosylation patterns have been connected to disease states and can impact the function of uromodulin in the kidneys [60,61]. More than 580 phosphorylation and 720 glycosylation sites have been found in important proteins involved in cellular transport and membrane integrity. Phosphoproteins and glycoproteins, like those found in rat kidney tissues, can display a variety of modification sites. Furthermore, concomitant phosphorylation and glycosylation in proteins such as fibronectin suggests that these PTMs may interact with the mechanisms underlying kidney disease [62,63,64].

Finding new kidney disease biomarkers has been made easier by proteomic techniques. In particular, urinary proteomics has proven to be an effective non-invasive method for identifying kidney disease in its early stages. For instance, certain urine protein signatures have been linked to DKD [65]. The urinary proteomic classifier CKD273 has shown promise not only in predicting the progression of CKD but also in forecasting cardiovascular outcomes in patients with CKD. In a cohort study, higher CKD273 scores were strongly associated with an increased risk of fatal or non-fatal cardiovascular events, especially in patients with early-stage CKD without a history of cardiovascular disease, where those in the highest CKD273 tertile had a more than tenfold increased risk compared to those in the lowest tertile [66]. The discovery of urinary exosome proteome analysis, a non-invasive approach for identifying disease-specific biomarkers and quantifying kidney damage, has allowed for the real-time monitoring of renal pathology. Proteins found in exosomes, which are small extracellular vesicles generated by kidney cells, reflect the condition of both healthy and sick kidneys. Notably, exosomal proteins such as aquaporin-1 (AQP1) and aquaporin-2 (AQP2) have been discovered as possible kidney disease biomarkers. For example, AQP2, an important membrane protein involved in water reabsorption, has been found in urine exosomes, offering information on water-balance issues. Exosomal proteomic profiles have additionally shown diagnostic utility in differentiating among several kidney diseases, including DKD. In order to identify novel biomarkers associated with the development of DKD, recent research has employed extensive urine and exosome proteomics analysis in people with type 2 diabetes and varying degrees of albuminuria. Additionally, urine exosomal microRNA profiling was utilized to differentiate DKD from other kidney diseases, including focal segmental glomerulosclerosis, highlighting the distinctiveness of exosomal contents in representing various kidney diseases [67,68,69,70].

Proteogenomics, which combines proteomics, transcriptomics, and genomics, has helped to identify new protein isoforms and mutations, such as alternative splicing events and protein-coding mutations, that contribute to the prevalence and severity of kidney illnesses. Alternative splicing is an important mechanism for diversifying the transcriptome and proteome, influencing many biological processes and disease states. Disruptions or mutations in the splicing pathway can interfere with the creation of mature mRNA and functioning proteins, leading to a range of illnesses. Furthermore, the alternative splicing of protein-coding messenger RNAs is a key regulatory mechanism in eukaryotic gene expression that ensures that proteins function properly [71,72].

### 2.4. Metabolomics

Metabolomics, or the comprehensive investigation of tiny molecules inside biological systems, has contributed to the identification of metabolic changes related with kidney disease. This technique improves renal diagnosis, management, and monitoring by allowing the identification of possible therapeutic targets, biomarkers, and disease causes. For example, the combined analysis of plasma proteome and transcriptome data has identified 32 possible therapeutic targets for CKD, opening up possibilities for new immunotherapies and targeted interventions. Metabolomic profiling has shown variations in metabolite profiles that can act as biomarkers for the early detection and monitoring of kidney illnesses, such as the identification of adenine as a critical marker of renal fibrosis in diabetic kidney disease [35,73]. Nuclear magnetic resonance (NMR) and high-resolution mass spectrometry are two new analytical tools that have increased the accuracy of metabolomic profiling. NMR spectroscopy is a non-destructive technique for detecting and measuring metabolites in complex biological materials. It provides detailed structural information and allows for the examination of intact tissues and biofluids. However, because of its high sensitivity and mass accuracy, excellent-resolution mass spectrometry can detect a wide range of metabolites, even at low concentrations. The combination of these methodologies has accelerated metabolomics research, allowing for a more in-depth understanding of metabolic changes linked with numerous diseases, including kidney problems [74]. These methods improve the accuracy of kidney disease biomarker identification by detecting a broader range of metabolites [23]. Metabolic research has provided insight into several aspects of kidney disease pathophysiology, such as abnormal energy metabolism and oxidative stress. It is assumed that these metabolic changes exacerbate kidney damage and fibrosis, two key pathological features that contribute to the development of CKD. The kidneys’ high density of mitochondria and substantial energy demands make renal tubular cells especially susceptible to mitochondrial dysfunction under stress conditions like hypoxia, contributing to CKD. Key metabolites, such as fumarate and NAD precursors, have been identified as markers of mitochondrial dysfunction, with fumarate linking oxidative stress to tissue fibrosis [75,76]. Niacinamide supplementation has exhibited renoprotective effects by increasing nicotinamide adenine einucleotide (NAD^+^) levels, indicating a viable therapeutic option. NAD^+^ deficiency in CKD is associated with mitochondrial impairment and cellular senescence. Niacinamide administration can restore NAD^+^ levels, potentially slowing disease development [75]. Understanding these disturbed pathways helps to reveal the underlying causes of disease and may lead to the development of novel, tailored therapeutics aimed at blocking these processes and slowing disease progression [25].

Lower levels of essential amino acids and higher levels of uremic toxins indicate significant abnormalities in amino acid metabolism in patients with CKD. This imbalance highlights how CKD affects protein metabolism and clearance, leading to the accumulation of toxic metabolites that can worsen symptoms and accelerate disease progression [77]. Furthermore, fluctuations in metabolite levels have been discovered as kidney disease advances, notably in DKD, utilizing temporal metabolomics. Notably, patients with DKD have shown changes in amino acid metabolism, including lower levels of tryptophan and its metabolites. These metabolic alterations are linked to increased oxidative stress and inflammation, which contribute to disease progression. The kynurenine pathway, which is responsible for tryptophan breakdown, generates metabolites that might influence immunological responses and oxidative stress. The increased activation of this system in DKD causes the accumulation of pro-inflammatory and pro-oxidative compounds, increasing kidney impairment. Furthermore, low tryptophan availability affects the production of serotonin and melatonin, which have antioxidant and anti-inflammatory characteristics, enhancing oxidative stress and inflammation. These findings highlight the significance of tryptophan metabolism in DKD pathophysiology and suggest that targeting this pathway could offer novel therapeutic strategies [78]. Furthermore, kidney fibrosis and reduced kidney function in DKD have been linked to lipid metabolic changes, particularly higher levels of some sphingolipids. Sphingolipids, including ceramides and sphingosine-1-phosphate (S1P), are bioactive chemicals that control biological processes such as cell death, proliferation, and inflammation. The dysregulation of sphingolipid metabolism can cause podocyte damage, glomerular sclerosis, and tubulointerstitial fibrosis, all of which are associated with DKD development. According to recent research, changes in sphingolipid levels can lead to oxidative stress, inflammatory reactions, and fibrotic processes, all of which contribute to renal failure. For instance, an increase in ceramide accumulation has been associated with renal cell death and mitochondrial dysfunction, both of which worsen kidney injury. Furthermore, increased S1P signaling has been related to renal fibrosis due to the activation of profibrotic pathways. These findings indicate that targeting sphingolipid metabolism may provide novel therapy methods for lowering kidney fibrosis and maintaining renal function in DKD [79,80,81]. These findings emphasize the utility of metabolomic profiling in developing biomarkers for early disease identification and DKD surveillance [82]. Urine metabolites have been associated with poor kidney outcomes, disease progression, and death in patients with CKD. These indications are particularly useful in identifying patients who are at risk of rapid disease development, allowing for earlier intervention and customized care strategies [83]. In addition to identifying individual metabolites, examining groups of similar compounds has identified certain pathways impacted by kidney disease. For instance, changes in lipid and amino acid metabolism have been brought to light by metabolomics, with branched-chain amino acids and their metabolites demonstrating strong correlations with CKD. These findings provide support for pathway-level targeting as a therapy strategy [84]. Lipidomics, a subfield of metabolomics focused on lipid profiles, has revealed that lipid metabolism abnormalities are frequent in CKD and glomerular disease. Changes in certain lipid species, such as ceramides, phosphatidylcholines, and cholesterol esters, have been associated with kidney damage, inflammation, and fibrosis, which are major causes of CKD development. Ceramide levels have been linked to increased oxidative stress and mortality in kidney cells, which leads to fibrosis. Similarly, aberrant phosphatidylcholine metabolism can worsen kidney injury by interfering with signaling pathways and compromising cell membrane integrity. Cholesterol ester accumulation has also been connected to inflammation and renal injury. These lipidomic alterations provide insights into the molecular mechanisms underlying CKD and highlight potential therapeutic targets for mitigating disease progression [80]. This result shows that lipidomics could be utilized to design lipid-targeted treatments, possibly focused on modifying these pro-inflammatory and pro-fibrotic pathways [24].

The gut microbiota has a profound impact on the metabolome, creating chemicals that may be associated with kidney disease. Two gut-derived uremic toxins, p-cresyl sulfate and indoxyl sulfate, have been linked to the advancement of CKD and its consequences for the cardiovascular system. Because of poor renal clearance, these gut-bacteria-produced protein-bound solutes build up in CKD. High levels of p-cresyl sulfate and indoxyl sulfate have been associated with increased oxidative stress and inflammation, which contribute to renal fibrosis and hasten CKD progression. Furthermore, larger concentrations of these toxins are associated with an increased risk of cardiovascular events and all-cause death in patients with CKD. Strategies for lowering p-cresyl sulfate and indoxyl sulfate levels, such as dietary changes, the use of oral sorbents such as AST-120, and gut microbiota manipulation, are being studied to attenuate their negative effects on renal and cardiovascular health [85,86]. The identification of microbiome-derived metabolites facilitates the understanding of the role of gut health in kidney disease as well as the potential benefits of microbiome-targeted treatments [23].

### 2.5. Epigenomics

Epigenomics is the comprehensive study of epigenetic alterations, which are heritable differences in gene expression that do not alter the DNA sequence. Single-cell epigenomic techniques such as ATAC-seq and methylome sequencing are used to investigate cell-type-specific epigenetic modifications in kidney tissues (Table 2). This strategy has greatly aided the discovery of discrete epigenetic profiles in specific kidney cell groupings, such as podocytes and tubular cells, that contribute primarily to disease states [87].

Acetylation and methylation are examples of post-translational modifications of histone proteins that influence chromatin structure and gene accessibility. Renal biopsies from patients with DKD have shown increased histone H3 lysine 9 acetylation (H3K9ac), which is associated with the increased production of profibrotic genes such as TGF-β1. Through the activation of histone acetyltransferases such as p300/CBP in mesangial cells, TGF-β1 has been demonstrated to induce histone acetylation in target gene promoters, including plasminogen activator inhibitor-1 (PAI-1) and p21 [88,89]. Targeting histone-modifying enzymes is a potential treatment strategy to slow the progression of renal disease [27]. DNA methylation, which involves adding methyl groups to cytosine residues, is critical for controlling gene expression. Methylation patterns have been linked to a variety of kidney diseases. For example, the hypermethylation of the Ras-association domain family 1 isoform A (*RASSF1A*) gene promoter has been linked to renal cell carcinoma, indicating that it plays a role in carcinogenesis. RASSF1A promoter methylation is associated with poorer cancer-specific survival in patients with RCC [90,91]. Furthermore, the advancement of CKD has been connected to the hypomethylation of particular genes, suggesting possible biomarkers for early diagnosis [26].

Beyond standard histone modifications, histone variants influence gene expression by modulating chromatin properties, impacting kidney disease progression. H2A.Z is a histone variation that acts as both a transcription activator and repressor. Its inclusion in nucleosomes can alter chromatin structure, thus influencing gene expression. H2A.Z plays an important role in a variety of cellular activities, including transcriptional control, DNA repair, and centromeric heterochromatin regulation. Cancer and other disorders have been associated with the deregulation of H2A.Z. The presence of H2A.Z in kidney cells may modify chromatin structure, influencing gene expression, especially during stress or sickness. However, few studies have directly linked changes in H2A.Z dynamics to modifications in gene expression in kidney cells under these conditions. Additional research is required to establish the precise involvement of H2A.Z in renal pathogenesis [87,92]. Recent research demonstrates how chromatin’s spatial organization influences gene expression in kidney disease by bringing enhancers and promoters into contact. Chromatin loops connect kidney-specific genes to distal regulatory elements, which influences gene regulation in CKD and nephropathy. Using chromatin conformation capture techniques, researchers have discovered links between CKD-associated susceptibility areas and the transcription start sites of 304 target genes, many of which are essential to kidney homeostasis. A geographically resolved human kidney multi-omics single-cell atlas has revealed insights into the fibrotic milieu in kidney illness, emphasizing the importance of spatial chromatin arrangement in disease development [93,94].

Environmental factors such as pollution, toxins, and lifestyle decisions can all cause epigenetic changes in kidney cells. These modifications, such as increased DNA methylation or histone acetylation, may accumulate as a result of exposure in susceptible individuals, notably in CKD and DKD, and contribute to disease progression [95].

Non-coding RNAs, including long non-coding RNAs (lncRNAs) and microRNAs, regulate gene expression post-transcriptionally. The dysregulation of microRNAs has been linked to kidney diseases. CKD is connected with increased miR-21 levels, which inhibit antifibrotic genes and induce fibrosis. MiR-21 promotes renal fibrosis by downregulating antifibrotic genes, including peroxisome proliferator-activated receptor alpha (PPAR-α), which leads to fibrotic processes. MiR-21 can activate Toll-like receptor 7 (TLR7) in renal epithelial cells, resulting in pro-inflammatory responses that worsen kidney fibrosis [96,97]. Similarly, DKD has been associated with lncRNAs such as MALAT1, which regulate the synthesis of extracellular matrix components and inflammatory responses. Long non-coding RNA metastasis-associated lung adenocarcinoma transcript 1 (MALAT1) is upregulated in DKD and contributes to disease progression by modulating extracellular matrix (ECM) synthesis and inflammatory responses. In high glucose-stimulated human renal tubular epithelial cells (HK-2), MALAT1 knockdown alleviated cell viability inhibition, apoptosis, and inflammatory responses, effects that were reversed by a miR-15b-5p inhibitor or TLR4 overexpression, indicating that MALAT1 promotes DKD progression via the miR-15b-5p/TLR4 signaling axis. MALAT1 is upregulated in the urine of patients with type 1 diabetes mellitus with DKD, suggesting its potential as a biomarker for the disease [98,99]. The potential of DNA methyltransferase and histone deacetylase inhibitors to reverse abnormal epigenetic changes and restore normal gene expression patterns (“epigenetic plasticity”) is currently under investigation, which may lead to new therapeutic options [100].

**Table 2:** Emerging technologies and computational approaches in omics integration for nephrology.

Technology/Approach	Description	Applications in Nephrology	Challenges	References
Single-Cell Omics	Analyzes molecular profiles at the single-cell level, capturing cellular heterogeneity in kidney tissues	Identifies the specific cell populations and pathways involved in diseases like lupus nephritis and DKD	High data complexity and dimensionality	[87]
Spatial Transcriptomics and Proteomics	Retains the spatial context of molecular data within tissue samples	Maps localized disease processes within nephron segments; identifies cell-type-specific regulatory elements in kidney tubular cells linked to disease risk	Integration with bulk omics data; requires advanced computational tools	[101]
Explainable AI	AI models designed to provide interpretable predictions	Enhances transparency in predictive models for disease progression and drug response	Limited availability of explainable AI tools in clinical practice	[95]
Temporal Multi-Omics Integration	Integrates omics data across disease stages to capture dynamic molecular changes	Tracks the progression of kidney disease, identifying biomarkers and key intervention points in early CKD	Resource-intensive; requires longitudinal data across disease stages	[102]
Pharmacogenomics	Studies genetic variation in drug response, predicting potential adverse reactions and efficacy	Tailors treatments for patients with genetic mutations affecting drug metabolism, e.g., immunosuppressants	High cost and ethical concerns	[103]
Causal Inference Models	Uses statistical methods to determine cause–effect relationships in multi-omics datasets	Identifies causal molecular interactions in kidney disease, improving the understanding of disease pathways and potential intervention targets	Computationally intensive; complexity in integrating diverse omics data	[104]

Abbreviations: AI, artificial intelligence; CKD, chronic kidney disease; DKD, diabetic kidney disease.

## 3. Integration of Multi-Omics Data

To fully understand kidney diseases, multi-omics data such as transcriptomics, proteomics, metabolomics, epigenomics, and genomics must be considered. This comprehensive approach encourages progress in precision nephrology by improving our understanding of complex biological mechanisms, developing new biomarkers, and identifying therapeutic targets.

Combining transcriptomic and genomic data has revealed that particular genetic variants in patients with CKD are associated with gene expression changes. Such analyses reveal regulatory networks and processes that contribute to disease progression as well as possible intervention areas. For example, the CKDGen Consortium effectively used GWAS and transcriptome data to identify novel genes linked with kidney function. Several genes, including *TENM2*, *COL4A3*, and *DCLK1*, have recently been linked to kidney health indices such as eGFR and tubulointerstitial fibrosis. The increased expression of protective genes, such as *TENM2* and *SNX30*, is positively correlated with a greater eGFR and lower fibrosis levels, whereas genes such as *DCLK1* are associated with fibrosis, which may help to clarify the mechanisms underlying kidney disease progression. Integrating kidney-specific eQTL and GWAS data provides regulatory insights into these gene expressions, suggesting prospective treatment targets [105]. Understanding the evolution of kidney disease requires the temporal integration of omics data across different disease phases. Time-series investigations on DKD have revealed that molecular profiles change dynamically as the disease progresses, with stage-specific alterations in gene expression linked to fibrosis, inflammation, and metabolic dysfunction. Such analyses uncover early biomarkers and potential intervention targets that could be harnessed to delay disease progression by addressing these active pathways at specific stages [106]. Spatial multi-omics offers insights into molecular changes within distinct kidney compartments, such as glomeruli and tubules, and enhances our understanding of compartment-specific pathology. In glomerulonephritis, spatial omics data have identified inflammation- and fibrosis-associated molecular changes specific to different kidney structures [101].

Multi-omics integration has resulted in the identification of novel biomarkers and therapeutic targets for renal disorders. Proteomics and metabolomics studies have identified proteins and metabolites linked with different phases of CKD, which could serve as biomarkers for early diagnosis and monitoring. Specifically, combining Mendelian randomization (MR) and proteome-wide MR (PWMR) analysis revealed associations between CKD and kidney function with unique metabolites and inflammatory proteins, such as 2-hydroxyoctanoate and N-acetylalliin, pointing to potential therapeutic pathways [107]. The inclusion of various populations in multi-omics research improves the discovery of novel molecular markers and genetic connections, which are especially relevant for discovering risk factors for *APOL1*-associated kidney disease. This approach is critical for developing therapeutics tailored to underrepresented groups in nephrology [95]. By connecting known pharmacological pathways to biological targets in kidney disease, multi-omics integration facilitates therapeutic repurposing. Omics-based methods help identify medications with potential nephroprotective effects by using genetic and proteomic features that align with kidney disease pathways. By employing cutting-edge research such as pathway mapping and molecular docking to find medications that can more successfully alter key kidney disease processes, this integration expedites the discovery of new treatments [108].

Machine learning (ML) has made it easier to analyze large multi-omics datasets, detect trends, and create predictive models for kidney disease development. ML techniques, such as random forest survival analysis, have been applied to improve CKD risk prediction by merging clinical and omics data, thereby enabling personalized treatment regimens [109]. Pattern recognition and the causal analysis of molecular interactions in kidney disease are further improved by computational techniques such as Bayesian inference, deep learning, and causal inference models, which go beyond machine learning [104].

## 4. Applications of Precision Medicine Using Omics

Omics technology can transform precision medicine in nephrology by improving diagnosis and driving personalized treatment plans (Table 3). Genomics enables the discovery of hereditary kidney disease subgroups with discrete molecular pathways, such as *APOL1* mutations [11], which guide transplant decisions, while medications like VX-407 target specific polycystic kidney disease variants. Transcriptomics increases diagnostic accuracy by identifying different gene expression profiles associated with diseases, including glomerulonephritis, as well as insights into podocyte failure, which contributes to glomerulosclerosis and presents therapy options for maintaining podocyte integrity [110]. Proteomics allows for the distinction of diseases like DKD from other glomerular disorders by identifying distinct protein signatures, including biomarkers such as fibronectin and laminin, which are associated with disease development [111]. Metabolomics further complements this by identifying early markers of kidney impairment, such as fumarate, which highlights mitochondrial dysfunction and oxidative stress as both markers and therapeutic targets in DKD [112]. Spatial omics, by preserving the spatial context of molecular expression within kidney tissues, reveals localized disease mechanisms in nephron segments, critical for developing CKD-specific therapies. Recent chromatin interaction studies have improved our understanding of 3D regulatory networks and how they relate to disease risk [113]. Single-cell omics provides new insights into the cellular heterogeneity of kidney tissue, pinpointing the individual cells and pathways implicated in pathologies such as lupus nephritis and providing a more complete picture of renal development [37,114]. Epigenomics contributes by exposing how changes in DNA methylation and histone acetylation affect gene expression, enabling more reversible and tailored approaches to treating diseases, including kidney fibrosis and inflammation [115]. Predictive models based on machine learning advance medicine by offering accurate estimations of CKD progression and treatment outcomes [116,117]. By tailoring pharmaceutical regimens to the unique genetic profile of each patient, pharmacogenomics improves medication safety and efficacy, particularly when treating patients with immunosuppressants that have limited therapeutic windows [118]. Despite these advancements, thorough validation through trials and longitudinal research is still necessary for the clinical use of omics-driven biomarkers to ensure efficacy and reliability.

Recent advances in precision medicine have highlighted the potential of omics technology to improve the treatment of CKD. Reznichenko et al. (2024) developed a kidney-centric molecular classification, marking a significant development in this field [119]. Four distinct molecular categories were identified using a self-organizing map (SOM) machine learning technique and the transcriptome profiling of kidney biopsies: CKD-Blue, CKD-Gold, CKD-Olive, and CKD-Plum. These categories outperform traditional CKD staging methods and histology diagnoses by capturing disease diversity and reflecting the underlying molecular pathways that drive progression. These informed molecular categories include a variety of biological processes that hint at potential therapeutic targets. For example, CKD-Gold has been connected to pro-inflammatory and profibrotic pathways, CKD-Plum to protein synthesis and vesicle transport, and CKD-Olive to increased mitochondrial activity. It is noteworthy that, among several patient groups, CKD-Gold had the highest incidence of progression to kidney failure, highlighting its predictive and therapeutic utility. Additionally, the study identified distinct patterns in the urine proteome associated with these molecular categories, paving the way for classification based on non-invasive biomarkers. With enhanced prediction accuracy and individualized therapy possibilities, this methodology enables a shift from existing systemic and indirect approaches to categorizing CKD through a more precise, tissue-specific molecular approach. The stability of the data across several cohorts underscores its therapeutic significance and scalability. This study demonstrates how precision medicine can provide valuable insights into disease processes, progression, and therapy options by integrating omics technology into the classification of CKD. This collaboration will advance the field of nephrology, ultimately improving patient outcomes.

## 5. Challenges and Future Directions

The use of omics technology in nephrology has greatly improved our understanding of kidney disease. However, various hurdles must be overcome before it can reach its full potential in clinical practice. The lack of defined formats and protocols across different omics platforms complicates the integration of multi-omics data. Due to differences in data types, volumes, and formats, merging various omics datasets, such as those of transcriptomics, proteomics, metabolomics, and genomics, poses significant challenges. Standardized procedures and modern computational tools are essential for effective integration, ensuring accurate and meaningful analysis. For instance, the use of multi-omics data to explore the renal medulla has underscored the need for advanced analytical tools to manage data complexity and heterogeneity [120].

Large, high-quality datasets are required for rigorous omics research to ensure statistical power and reproducibility. However, these numbers are frequently restricted, particularly for certain kidney diseases or underrepresented groups. The creation of extensive repositories that can facilitate significant research, cooperation, and data-sharing programs is crucial. The German Chronic Kidney Disease (GCKD) initiative [121] underlines the value of large-scale datasets in detailed analysis [2].

Single-cell and spatial omics approaches generate high-resolution data at the cellular level, providing important insights into kidney disease. However, because of the data’s great complexity and intricate structures, these technologies make data analysis even more difficult. Accurately capturing cellular variability within kidney tissues requires the development of algorithms and techniques for combining single-cell and spatial data with bulk omics, which is a continuous problem [101]. Advanced computational tools, especially ML and AI, are used to manage and analyze complex multi-omics data. However, interpreting these models, especially in clinical contexts, is challenging. Explainable AI approaches are needed to improve transparency in predictions and model decisions, allowing healthcare providers to understand the basis of these results and enhancing physician and patient trust in omics-based models.

Although omics technologies have revealed multiple potential indicators for kidney disease, converting them into clinically proven tools remains difficult. Kidney diseases occur often, making longitudinal data important for investigating molecular changes over time. However, gathering and integrating time-series omics data necessitates significant resources and collaboration across study sites. Longitudinal studies have the potential to identify crucial intervention points, but they are neglected due to logistical and financial constraints. Increasing resources and financial possibilities to promote longitudinal omics investigations in nephrology would allow for earlier and more effective interventions [102].

Rigorous validation studies are required to confirm the relevance and reliability of the biomarkers in a wide range of individuals and environments. Furthermore, regulatory constraints may make it difficult to approve omics-based biomarkers, thus delaying the clinical use of promising candidates. These technologies may be inaccessible because of the high cost of omics analysis, especially in healthcare settings with limited financing. This topic emphasizes how important it is to develop affordable omics solutions to ensure that discoveries benefit a larger population. To integrate omics technologies into standard nephrology practice and enable fair access to these innovations, costs must be decreased and accessibility increased.

Also, ethical concerns are significant, specifically around privacy, data ownership, and genetic bias. In particular, genetic and epigenetic data present ethical issues, including the potential for unforeseen consequences and their effect on a patient’s family. Patients must understand the extent and ramifications of omics testing, including the effects on family members, in order to use data responsibly. One example of a change in policy that highlights the importance of moral frameworks that uphold justice and safeguard patient rights is the removal of race-based algorithms from kidney function testing.

Despite these challenges, integrating omics data has the potential to lead to novel therapeutic breakthroughs. Omics techniques can help discover new therapeutic targets and guide the creation of tailored treatments by clarifying the molecular pathways behind kidney disorders. Multi-omics integration, for example, has been critical in elucidating the intricacies of kidney disease and paving the way for targeted therapeutics.

## Figures and Tables

**Table 1:** Key omics technologies and their applications in kidney disease research.

Omics Technology	Main Focus	Applications in Kidney Disease	Examples of Insights	References
Genomics	Study of genetic variations	Identifies gene mutations linked to monogenic kidney disorders and CKD risk genes; assesses polygenic risk for kidney disease	Mutations in *PKD1* and *PKD2* in ADPKD; *APOL1* variants associated with CKD susceptibility in African populations; GWAS loci linked to CKD progression	[16,18]
Transcriptomics	Analysis of RNA expression	Characterizes disease-specific gene expression patterns and cellular interactions; tracks cell-type-specific responses in kidney disease	scRNA-seq identifies immune cell subsets in lupus nephritis; novel biomarkers for podocyte failure in glomerulosclerosis	[19,20]
Proteomics	Protein expression and modifications	Identifies biomarkers, post-translational modifications, and protein interactions relevant to kidney disease progression	Urinary proteomics classifier CKD273 predicts CKD progression and cardiovascular risk; altered glycosylation of uromodulin in CKD	[21,22]
Metabolomics	Analysis of small molecules	Identifies metabolic signatures and potential biomarkers for the early detection and progression of kidney diseases	Increased uremic toxins in CKD; fumarate and NAD precursors indicating mitochondrial dysfunction in CKD; lipidomic findings on ceramides and sphingolipids linked to kidney fibrosis	[23,24,25]
Epigenomics	Epigenetic modifications	Examines DNA methylation, histone modifications, and chromatin structure; evaluates treatment response and disease progression	TGF-β1 promoter acetylation linked to kidney fibrosis in DKD; hypomethylation as a biomarker for early CKD detection; spatial chromatin looping linked to nephropathy genes	[26,27]

Abbreviations: ADPKD, autosomal polycystic kidney disease; APOL, apolipoprotein; CKD, chronic kidney disease; DKD, diabetic kidney disease; NAD, nicotinamide adenine dinucleotide; PDK, polycystic kidney disease; PRS, polygenic risk scores; RASSF1A, Ras-association domain family member 1 isoform A; TGF, transforming growth factor.

**Table 3:** Applications of precision medicine using omics in nephrology.

Omics Approach	Unique Findings	Applications	References
Genomics	Identification of genetic mutations like *APOL1* risk alleles.	Guiding transplant decisions and targeted treatments such as VX-407 for PKD1 mutations.	[11]
Transcriptomics	Insights into podocyte mitotic catastrophe linked to glomerulosclerosis.	Identifying therapeutic targets to maintain podocyte integrity and delay glomerular disease progression.	[110]
Proteomics	Biomarkers like fibronectin and laminin associated with DKD progression.	Differentiation of DKD from other kidney diseases, and the development of individualized diagnostic tools.	[111]
Metabolomics	Fumarate identified as a mediator of oxidative stress and mitochondrial dysfunction in DKD.	Early diagnosis of kidney disease, and targeting energy metabolism disturbances for therapeutic interventions.	[112]
Spatial Omics	Localization of disease processes in specific nephron segments using spatial transcriptomics.	Tailored therapy in CKD by understanding segment-specific mechanisms and chromatin architecture.	[113]
Single-cell Omics	Discovery of new renal cell types and pathways in lupus nephritis.	Uncovering cell-specific therapeutic targets and cellular lineage interactions for better diagnostics and therapy.	[37,114]
Epigenomics	Role of DNA methylation and histone deacetylase inhibitors in reversing gene expression changes.	Reversible modulation of renal fibrosis and inflammation, enabling tailored treatments.	[115]
Pharmacogenomics	Study of genetic variations influencing drug metabolism and immunosuppressant response.	Reduction in adverse drug reactions, and personalization of polypharmacy regimens.	[118]

## Data Availability

No new data were created or analyzed in this study.
